# Vedolizumab in Mild-to-Moderate Crohn’s Disease Patients Naïve to Biological Therapy: A Multicentric Observational Study

**DOI:** 10.1093/crocol/otad053

**Published:** 2023-09-22

**Authors:** Adriana Zanoni Dotti, Daniela Oliveira Magro, Eduardo Garcia Vilela, Julio Maria Fonseca Chebli, Liliana Andrade Chebli, Flavio Steinwurz, Marjorie Argollo, Nayara Salgado Carvalho, Jose Miguel Luz Parente, Murilo Moura Lima, Rogério Serafim Parra, Ramir Luan Perin, Cristina Flores, Eloá Marussi Morsoletto, Sandro da Costa Ferreira, Juliano Coelho Ludvig, Roberto Luiz Kaiser Junior, Mikaell Alexandre Gouvea Faria, Guilherme Mattioli Nicollelli, Adriana Ribas Andrade, Natalia Sousa Freitas Queiroz, Paulo Gustavo Kotze

**Affiliations:** Hospital de Clínicas das UFPR, Curitiba, Brazil; Universidade Estadual de Campinas (UNICAMP), Campinas, Brazil; Faculdade de Medicina da Universidade Federal de Minas Gerais, Belo Horizonte, Brazil; Universidade Federal de Juiz de Fora, Brazil; Universidade Federal de Juiz de Fora, Brazil; Hospital Israelita Albert Einstein, São Paulo, Brazil; Hospital São Luiz (Rede D’or), São Paulo, Brazil; Hospital São Luiz (Rede D’or), São Paulo, Brazil; Universidade Federal do Piauí, Teresina, Brazil; Universidade Federal do Piauí, Teresina, Brazil; Hospital das Clínicas da Faculdade de Medicina de Ribeirão Preto, Universidade de São Paulo, Brazil; Universidade de Passo Fundo (UPF), Passo Fundo, Brazil; Instituto do Aparelho Digestivo (IAD), Porto Alegre, Brazil; Hospital São Vicente, Curitiba, Brazil; Hospital das Clínicas da Faculdade de Medicina de Ribeirão Preto, Universidade de São Paulo, Brazil; Clínica LUDVIG/ ESADI, Blumenau, Brazil; Kaiser Clínica, São José do Rio Preto, Brazil; Kaiser Clínica, São José do Rio Preto, Brazil; Hospital de Clínicas das UFPR, Curitiba, Brazil; Universidade Estadual da Bahia (UNEB), Salvador, Brazil; Hospital Santa Cruz, Curitiba, Brazil; Pontificia Universidade Católica do Paraná (PUCPR), Curitiba, Brazil

**Keywords:** Crohn disease, inflammatory bowel diseases, biological therapy

## Abstract

**Background:**

In real-world experience, the number of patients using vedolizumab as first-line biological therapy was low. We aimed to evaluate the effectiveness and safety of vedolizumab in mild-to-moderate Crohn’s disease (CD) biologic-naïve patients.

**Methods:**

We performed a retrospective multicentric cohort study with patients who had clinical activity scores (Harvey–Bradshaw Index [HBI]) measured at baseline and weeks 12, 26, 52, as well as at the last follow-up. Clinical response was defined as a reduction ≥3 in HBI, whereas clinical remission as HBI ≤4. Mucosal healing was defined as the complete absence of ulcers in control colonoscopies. Kaplan–Meier survival analysis was used to assess the persistence with vedolizumab.

**Results:**

From a total of 66 patients, 53% (35/66) reached clinical remission at week 12. This percentage increased to 69.7% (46/66) at week 26, and 78.8% (52/66) at week 52. Mucosal healing was achieved in 62.3% (33/53) of patients. Vedolizumab was well tolerated, and most adverse events were minor. During vedolizumab treatment, 3/66 patients underwent surgery.

**Conclusions:**

This study demonstrates the effectiveness and safety of vedolizumab as a first-line biological agent in patients with mild-to-moderate CD.

## Graphical Abstract

**Graphical Abstract:**
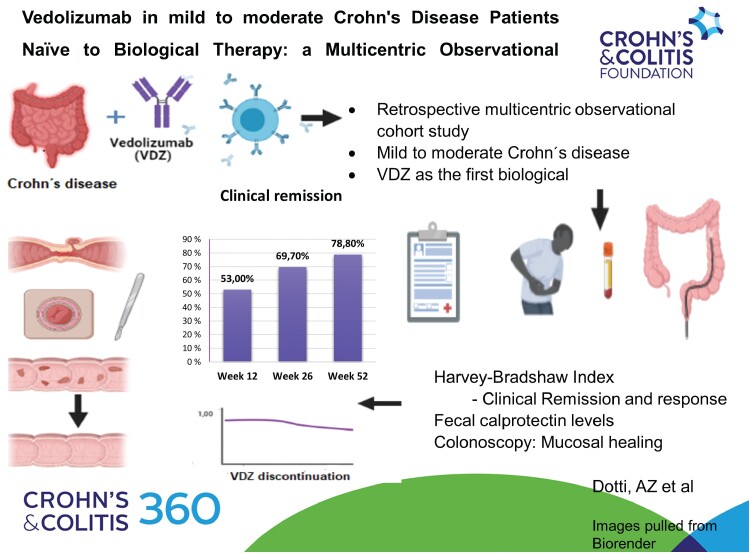

## Introduction

Crohn’s disease (CD) is characterized as a chronic, idiopathic, and immune-mediated disease, which can affect individuals at any age with significant morbidity and impact on their quality of life.^1^ As the precise etiology of CD remains unknown, curative therapy is not yet a reality. Aiming disease control, conventional therapy options include corticosteroids and immunomodulators such as azathioprine, 6-mercaptopurine, or methotrexate. One of the major advances in the management of CD was comprised of the approval of biological agents. This class of medications includes antitumor necrosis factor (anti-TNF) agents (infliximab, adalimumab, and certolizumab pegol), anti-integrins (vedolizumab), and anti-interleukins (ustekinumab). All biological agents are effective in the treatment of CD as the first option, or as second line, in patients previously exposed to other biological agents.^2^

With a distinct therapeutic target, the class of anti-integrin antibodies has recently expanded. Vedolizumab is a human IgG α4-β7 inhibitor monoclonal antibody with gut-specific properties, which blocks leucocyte trafficking from the endothelium to the intestine. Its mechanism of action comprises blocking the interaction between the α4-β7 integrin in lymphocytes and the MAD-CAM1 adressin at the endothelial wall. Consequently, lymphocyte trafficking is blocked, and a reduction of the population of inflammatory cells in the different layers of the intestine is achieved.^3^

GEMINI II and III, randomized clinical trials, demonstrated the efficacy and safety profile of vedolizumab in the induction and maintenance of clinical remission and response in patients with CD. Both studies included patients with moderate-to-severe CD, naïve to antitumor necrosis factor (anti-TNF) inhibitors, or who had inadequate response, loss of response, or intolerance to anti-TNF agents.^4,5^

Because vedolizumab was approved by regulatory agencies, several real-life cohort studies and meta-analyses describing the effectiveness and safety of the drug have been published, both in naïve and in patients previously exposed to other biologicals.^6–14^

Response to vedolizumab may vary according to not only previous therapies but also to disease characteristics. To overcome this issue, specific clinical decision tools have been developed to identify patients who may present better response to the agent in clinical practice. A study from the GETAID could identify through one of these tools which patient profile could benefit from dose optimization and an extra dose of vedolizumab at week 10, with a higher probability of clinical response and avoidance of surgery.^15^ Another American study evaluated if this tool (which used 5 main variables: previous surgery, fistulizing CD, previous anti-TNF, serum albumin, and C-reactive protein level) could predict utilization of healthcare resources in CD patients under vedolizumab therapy.^16^ This strategy could improve the identification of patients who would have better response rates before treatment initiation, with bio-naïve patients, with no previous surgery, and fistulizing disease possibly being better-predicted candidates for improved outcomes.

However, despite the development of these prediction tools, there is a scarcity of data regarding the use of vedolizumab as the first biological agent option, mostly in mild-to-moderate CD. This study aimed to evaluate the effectiveness of vedolizumab exclusively in CD patients who were naïve to previous biologics, with mild-to-moderate disease. Additionally, we aimed to analyze the safety profile of vedolizumab, rates of mucosal healing, need for abdominal surgery, and drug discontinuation over time in this specific population.

## Methods

This was a retrospective multicentric observational cohort study, with patients with mild-to-moderate CD who used vedolizumab for their treatment as the first biological agent at any time during their disease course.

The study was carried out with a convenience sample, with patients captured from different inflammatory bowel disease tertiary referral centers in Brazil. The period of inclusion of data was from August 2021 to May 2022, but patients could have initiated vedolizumab at any time of their disease course since drug approval and reimbursement in Brazil. Indications for vedolizumab were based on active CD with failure to conventional therapy. We included adult patients with an established diagnosis of CD with clinical, endoscopic, serologic, radiographic, and/or histologic criteria, who were treated with vedolizumab for at least 12 weeks, on an outpatient basis, and were refractory to conventional treatment (steroids and/or immunomodulators such as azathioprine and methotrexate), with no previous use of any biological agent. Patients were excluded in case of any of the following criteria: severe CD (Harvey–Bradshaw index [HBI] > 16); prior exposure to anti-TNF inhibitors or ustekinumab; indeterminate colitis; patients admitted to the hospital; pregnant and pediatric patients.

All patients used standard doses of vedolizumab (300 mg intravenous at weeks 0, 2, and 6 as induction), followed by a maintenance dose of 300 mg every 8 weeks, as approved in our country. At physicians’ discretion, patients could have an additional dose at week 10, and in cases of possible primary nonresponse, partial response, or secondary loss of response, dose optimization with 300 mg every 4 weeks could be prescribed.

### Study Variables

Demographic and clinical data were collected from electronic medical charts. The following variables were analyzed: age at vedolizumab induction, disease duration from diagnosis to treatment initiation, gender, body mass index, smoking history, concomitant or previous medications (corticosteroids, immunomodulators), previous abdominal surgeries, and disease characteristics according to the Montreal classification.

Clinical assessments were obtained retrospectively from outpatient consults’ electronic medical records from weeks 12, 26, and 52 and at the last follow-up using the HBI to assess clinical response and remission. This index is used routinely in all centers for all CD-related outpatient consults, at all visits. We also analyzed adverse events, rates of mucosal healing, the need for major abdominal surgery during treatment, and vedolizumab discontinuation. Calprotectin levels, whenever available, were checked at each time point of the study. Rates of primary nonresponse, secondary loss of response, discontinuation of vedolizumab, and need for dose optimization or switch to a different biological agent were also checked.

### Definitions

For inclusion, mild-to-moderate CD was defined as an HBI between 5 and 16 points (5–7 as mild and 8–16 as moderate disease). Clinical remission was defined as an HBI ≤ 4 points, and clinical response was defined as a reduction in the HBI of 3 or more points from baseline. Mucosal healing was defined as a complete absence of ulcers in control colonoscopies, which could be performed in different time periods after induction, according to physicians’ discretion. Radiologic remission was defined as an absence of contrast enhancement in patients with previous active disease diagnosed by the same cross-sectional imaging method (magnetic resonance imaging [MRI] or computed tomography [CT] enterography). The last follow-up was defined as the last patient visit or the date of the last vedolizumab infusion in cases of treatment interruption. Primary nonresponse was defined as an HBI greater or equal to baseline after 16 weeks. We considered secondary loss of response patients with initial response who needed one of the following: dose optimization, abdominal surgery, or switch to a different biological agent.

### Ethical Aspects

This study protocol was registered on the clinicaltrials.gov website under the number NCT04362735. The study was also approved by the Institutional Review Board from the Catholic University of Paraná (PUCPR) with reference number 70875317.5.0000.0020, and the Federal University of Paraná (reference number 70875317.5.2001.0096). The study protocol was additionally approved by research ethical boards from each involved institution.

### Statistical Analysis

Efficacy data were captured in an “as observed” (denominators comprised only of patients who achieved the different time points of the study) and nonresponder imputation (NRI) (denominators of all patients in all time periods) analyses. Categorical variables were expressed as proportions and compared using chi-square or Fisher exact tests, where appropriate. Continuous variables were summarized using mean values, standard deviation, median values, and interquartile ranges. Kaplan–Meier curves were generated for time-to-event data (time until discontinuation of vedolizumab in months) and the need for surgery during follow-up. Differences in calprotectin levels were calculated with the nonparametric Wilcoxon test. We used IBM SPSS Statistics for Windows, version 20.0 (IBM Corp, Armonk, NY). The significance level adopted for the statistical tests was 5%.

## Results

A total of 72 patients were initially identified and 5 patients were excluded for having severe CD. One patient was excluded from the effectiveness analysis as the follow-up period was shorter than 12 weeks and was considered for the safety analysis exclusively. Overall, 66 patients were finally included for the effectiveness analysis at week 12, 64 patients at week 26, and 59 patients at week 52, with a median follow-up period of 24.5 months (interquartile range [IQR]: 14.75–40.00).

The clinical and demographic characteristics of included patients are detailed in Table 1. As observed, our patient population was mostly comprised of middle-aged individuals, nonsmokers, with a predominance of inflammatory and stricturing phenotype (Montreal B1 and B2), and disease duration greater than 24 months (*n* = 43; 65%). Overall, 65.2% of patients had moderate CD, and half of the patients had deep ulcers at baseline colonoscopy. Sixteen patients (24.2%) had previously undergone abdominal surgical procedures, and perianal disease was present in only 6% of patients (*n* = 4).

**Table 1.** Baseline characteristics of included patients.

Variable	
Age—years, median (IQR)	48 (32.5–65.8)
Female sex (*n*, %)	34 (51.5)
BMI [weight(kg)/height(m^2^)] (mean ± SD)	24.40 ± 4.57
Disease duration—months, median (IQR)	36 (12-84)
Current smoker (*n*, %)	5 (7.6)
Previous corticosteroids (*n*, %)	45 (68.2)
Previous azathioprine/methotrexate (*n*, %)	22 (33.3)
Montreal classification A (*n*, %)	
1	8 (12.1)
2	29 (43.9)
3	29(43.9)
Montreal classification L (*n*, %)	
L1	24 (36.4)
L2	19 (28.8)
L3	19 (28.8)
L4	4 (6.0)
Montreal classification B (*n*, %)	
B1	44 (66.7)
B2	17 (25.7)
B3	5 (7.5)
Perianal CD (*n*, %)	4 (6.0)
Previous abdominal surgery (*n*, %)	16 (24.2)
Disease activity before vedolizumab induction (*n*, %)	
Mild	23 (34.8)
Moderate	43 (65.2)
Deep ulcers at baseline colonoscopy (*n*, %)	33 (50.0)

Abbreviations: BMI, body mass index; CD, Crohn’s disease; IQR, interquartile range; SD, standard deviation.

**Figure 1.**
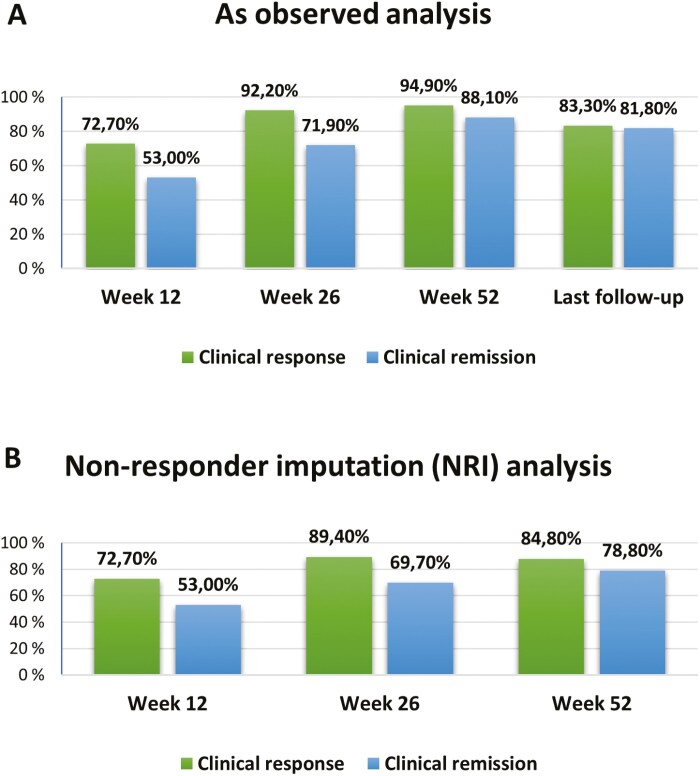
Efficacy data (clinical response and remission) at different periods: (A) as observed analyses; (B) nonresponder imputation analyses.

Regarding the primary outcome of the study, in “as observed” analysis, clinical remission was observed in 53% (35/66) of patients at week 12, 71.9% (46/64) at week 26, 88.1% (52/59) at week 52, and 81.8% (54/66) at the last follow-up visit. Clinical response was achieved in 72.7% (48/66), 92.2% (59/64), 94.9% (56/59), and 83.3% (55/66) in the same periods, respectively. In an NRI analysis, using 66 patients as denominators for all time periods, clinical remission was observed in 53.0% at week 12, 69.7% at week 26, and 78.8% at week 52. Clinical response was observed in 72.7%, 89.4%, and 84.8% in the same periods. These data are illustrated in Figure 1A and B.

**Figure 2.**
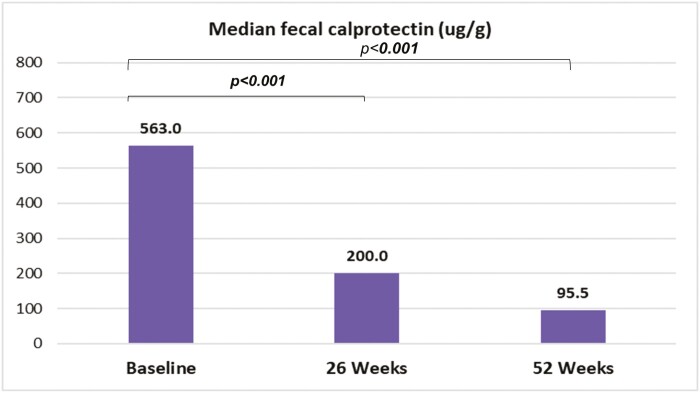
Median fecal calprotectin levels during follow-up after vedolizumab therapy. Significant values (*P* < .001) for 26 and 52 weeks when compared to baseline.

At the induction phase, an additional week 10 dose of 300 mg was administered in 15 patients (22.7%), and 45/66 patients (68.2%) were using corticosteroids. Combination therapy (vedolizumab + azathioprine or methotrexate) was used in 22 patients (33.3%). As demonstrated in Figure 2, there was a significant reduction in median fecal calprotectin levels during vedolizumab treatment: baseline (563 μg/g; IQR 315–1135); 26 weeks (200 μg/g; IQR 84.5–501.5; *P* < .001) and 52 weeks (95.5 μg/g; IQR 31.5–331; *P* < .001).

Overall, 6/66 (9.1%) patients were considered primary nonresponders, and secondary loss of response was observed in 14/66 (21.2%) patients. Optimization to 300 mg every 4 weeks, as maintenance therapy, occurred in one-third of patients (22/66—33.3%), including the 6 patients considered as primary nonresponders by HBI, 14 patients with secondary loss of response, and 2 who optimized to improve partial response. Overall, 8/14 patients with secondary loss of response improved, and continued vedolizumab therapy. In our study, 14 patients (21.2%) switched vedolizumab for another biological therapy (8 to ustekinumab, 4 to infliximab, and 2 to adalimumab), due to primary nonresponse (*n* = 6; 9.1%) or lack of efficacy (*n* = 8; 12.1%), both despite optimization.

**Figure 3.**
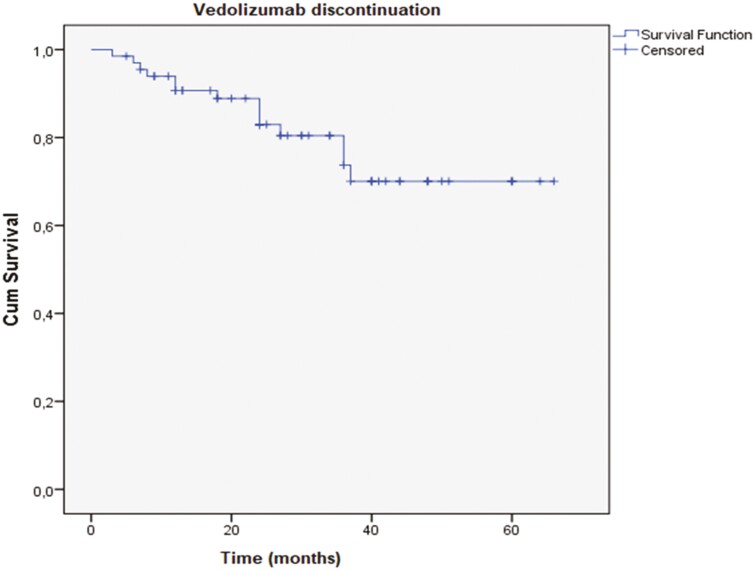
Kaplan–Meier curve with survival analysis for discontinuation of vedolizumab over time.

Figure 3 describes the persistence of vedolizumab use over time (time to event, in months, considering vedolizumab discontinuation). The cumulative survival curve for treatment discontinuation over time demonstrated that the probable period was 53.19 ± 2.97 months (95% confidence interval [CI]: 47.38–59.01).

Baseline colonoscopy findings were available in 64 patients, and active ulcerations were described in half of the patients (50.0%). A total of 53 patients underwent a control colonoscopy after vedolizumab initiation, in a mean period of 17.60 ± 27.36 months. Mucosal healing (complete absence of mucosal ulceration) was observed in 33/53 patients (62.3%).

At baseline, 63 patients had disease activity evaluation with cross-sectional imaging methods (CT or MRI enterography) and 52/63 (82.5%) had active disease (contrast enhancement) of affected intestinal segments. Post-treatment imaging evaluation methods were performed in 46 patients, with radiological remission (absence of contrast enhancement) in 21 (45.7%).

**Figure 4.**
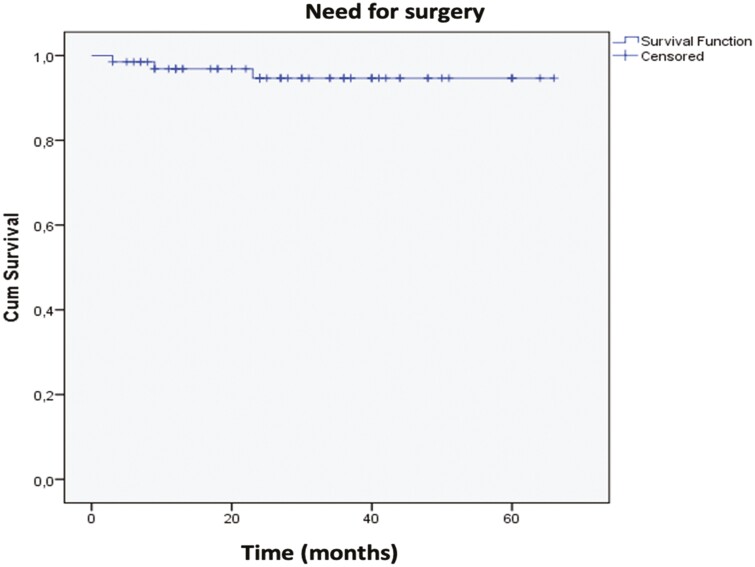
Kaplan–Meier curve with time to surgery as the main event.

During vedolizumab treatment, 3 patients underwent surgery (small bowel resection [*n* = 1]; ileocolonic resection [*n* = 1], and total colectomy with ileostomy [*n* = 1]). Figure 4 demonstrates the cumulative survival curve for abdominal surgery over time. The median time to surgery was 9.0 months ±4.9 (95% CI: 7.40–10.60).

For safety analysis, the patient with less than 12 weeks of follow-up was considered. Overall, 9/67 patients (13.4%) developed noninfectious adverse events. In detail, the following adverse events were reported: Nonspecific symptoms in 6 patients (headache, fatigue, arthralgia), alopecia in 2 cases, and 1 case of an infusion reaction.

Eighteen patients (26.8%) developed an infectious adverse event during vedolizumab treatment. The most common infectious event was upper respiratory tract infection (*n* = 6). *Clostridioides difficile* infection was observed in 3 patients, a urinary tract infection occurred in 1 patient, and 2 patients had COVID-19 (1 with a mild condition and the other with hospitalization). There was 1 case of gastroenteritis, 1 of osteomyelitis, and 1 central nervous system abscess requiring surgical drainage. There were no deaths during the study period.

## Discussion

This Brazilian multicentric study demonstrated the efficacy and safety profile of vedolizumab exclusively in naïve patients with mild-to-moderate CD. Clinical remission was observed in 69.7% of included patients after 6 months and 78.8% after 1 year of treatment, in the NRI analysis. Over the years, several real-life studies have demonstrated the effectiveness of vedolizumab in CD, confirming the results of pivotal studies.^5–9^ Most of these studies were performed in a more refractory population (moderate-to-severe CD), mainly consisting of patients with previous failure to other biologics, with a lower proportion of naïve patients. This multicentric Brazilian study demonstrated one of the first real-world evidence focused exclusively on evaluating the role of vedolizumab as the very first biological agent in mild-to-moderate CD, after failure to conventional therapy.

The effectiveness of vedolizumab in biologically naïve patients in our study was similar to what was previously described in some real-world multicentric studies, despite different patient populations.^11–14,17–19^ After induction, Macaluso et al. reported clinical response in 68.2% of patients with CD treated with vedolizumab at week 14.^18^ Kopylov et al. reported at the same period that 42/50 (82%) patients responded and 32 (64%) were in clinical remission.^14^

In the maintenance phase, the Italian cohort reported clinical response in 77.4% of CD patients at week 52.^18^ Kopylov et al., at the last follow-up period (44 weeks; IQR: 30–52 weeks), described that 27/35 (77.1%) patients responded to treatment and 24/35 (68.6%) were in clinical remission.^14^ A recent meta-analysis focused in bio-naïve patients demonstrated that clinical remission at week 52 was achieved in approximately 60% of CD patients.^19^ The remission rates from our study were higher as compared to these previous publications (clinical remission in 78.8% at week 52 and clinical response in 84.8%, in the same period—NRI analysis). A possible explanation for these findings is that, due to our inclusion criteria, our cohort had a significant proportion of patients with mild disease (34.8%), with no cases of severe disease. Moreover, there were slight differences in follow-up periods in some studies as compared to ours. There were also some differences in outcome definitions between our study and previously published data.

Data from the GEMINI trials and post hoc analyses indicated a remission rate of 48.9% after 12 months in the anti-TNF naïve population.^4,5^ Overall, the efficacy in retrospective real-world studies seems to be higher than in pivotal randomized controlled studies. The main reason for these findings is based on the absence of strict inclusion criteria in patient selection, in proper definitions of response and remission, and in the way missing data are considered in real-world data. These are possible reasons to justify the discrepancy of our efficacy data in comparison to the GEMINI studies in CD apart from the fact that the pivotal GEMINI trials were performed in patients with moderate-to-severe CD.

On the other hand, our rates of primary nonresponse, secondary loss of response, need for dose optimization, and drug discontinuation were similar to previously published similar studies, performed in a biologic-naïve population.^14,18^ In the study by Mohamed et al., although the rates of loss of response and discontinuation of vedolizumab were similar to ours, the rates of surgery during treatment were higher. These authors reported that 6 patients (24.0%) required an ileocecal resection, and 2 (8.0%) needed other procedures, such as a proctocolectomy and a segmental colectomy.^20^ The small proportion of the need for surgery in our study (3 patients) needs to be interpreted with caution, due to our limited sample size and the lack of more severe patients. We observed 1 small bowel resection, 1 ileocecal resection, and 1 total colectomy with ileostomy. Similarly, Macaluso et al. reported that only 4 CD patients underwent surgery (CD: 4.5%).^18^ More data are needed to assess whether low rates of surgery after the onset of vedolizumab in bio-naive patients are directly linked to treatment success or are affected by confounding factors, such as selection bias of included patients with milder disease.

In our study, mucosal healing was observed in 33/53 (62.3%) patients, in a mean period of 17.60 months after treatment initiation. Data from a meta-analysis revealed that rates of mucosal healing in bio-naïve patients with CD were 41.0% (95% CI: 9.0–77.0; *I*^2^ = 80.0%) at week 52.^19^ In the study from Macaluso et al., mucosal healing was observed in 41.9% (mucosal healing—Simple Endoscopic Score for CD [SES-CD] ≤2 for CD).^18^ The differences in mucosal healing rates between studies can be explained by the different definitions for endoscopic remission. In our study, we subjectively defined endoscopic healing as a complete absence of ulcers on colonoscopy and did not use objective endoscopic scores. In clinical practice, colonoscopy assessment is not based on validated endoscopic scores in most centers, which limited the use of an objective measurement in our cohort of patients.

In our study, optimization to 300 mg every 4 weeks, as maintenance therapy, was performed in 22/66 (33.3%) patients. A primary nonresponse (according to HBI) was observed in 6/66 (9.1%) cases, and a secondary loss of response was described in 14/66 (21.2%) patients. In a real-world multicentric study, with a similar methodology and population, similar numbers were described: A total of 22/79 (27.8%) patients underwent treatment optimization. The same study demonstrated similar rates of primary nonresponse (6 patients; 6.8%) and secondary loss of response (17 patients; 20.4%).^18^ Dose optimization for 300 mg every 4 weeks is recommended in patients with secondary loss of response, and recaptured response can be achieved after optimization in a significant proportion of patients.^21^

In our cohort, vedolizumab discontinuation was observed in 14/66 (21.2%) patients due to primary or secondary loss of response after dose optimization. In a multicentric European study, treatment was discontinued in 8 patients (14%) due to primary failure and adverse events.^14^ Our cohort demonstrated that the probability of continuing vedolizumab therapy, in a Kaplan–Meier survival analysis, was similar to what was observed in a study with a similar methodology.^22^ Patel et al., in another study in biologic-naïve patients, a greater treatment persistence with vedolizumab was observed over 12 months (85%).^23^ Some differences could be explained by a shorter mean duration of disease in this study, as compared to ours (4.4 vs 6.5 years) and by the percentage of patients undergoing surgery before treatment with vedolizumab (13.6% vs 24.2%).

In the GETAID study, clinical remission and response rates at week 14 were significantly higher in patients with higher and intermediate probability of response as compared to low probability (*P* = .04 and *P* = .045, respectively). The same tendency was observed at weeks 22 and 30 for clinical and steroid-free remission.^15^ These findings were in tune with our study, as our sample of patients was mostly composed of individuals with a high probability of response, which could explain our significant response and remission rates in all study periods. In the VICTORY consortium, patients with a low probability of response had lower endoscopic remission rates and a higher proportion of the need for surgery.^15^ In our study, only 2 patients had a low probability of response according to this prediction tool, which may limit comparison with previous analyses.

Our study demonstrated favorable safety outcomes, with 9 patients (13.4%) developing noninfectious adverse events, none of them being serious with no need for vedolizumab discontinuation. Eighteen patients (26.9%) developed infections and the most common was upper respiratory tract, followed by *C. difficile* infection. No malignancies were observed. Our outcomes of safety are in line with pivotal studies.^4,5^ Similarly, in a meta-analysis, 26.0% (95% CI: 16.0–36.0, *I*^2^ = 34%) of bio-naïve patients experienced adverse events to vedolizumab, and 4.0% (95% CI: 0.0–12.0, *I*^2^ = 0%) experienced serious adverse events.^17^ No new safety signals were observed in our cohort, in comparison with previously published data. If biologically naïve CD patients with mild-to-moderate disease present a different safety profile as bio-experienced, more severe, or UC patients, this still needs to be proved.

Our study is associated with some limitations that need to be outlined. This was a multicentric and retrospective study, with intrinsic methodology-related difficulties in data collection and possible slight differences in treatment strategies, despite using the same protocols. Our population was mostly comprised of less-refractory patients, with perianal disease in only 6.0% of the cases and 34.8% presenting mild disease at induction. This is a possible selection bias, as in clinical practice, patients with a more severe profile were probably treated with anti-TNF agents as the first option. One could question if mild CD is an off-label indication for biological therapy. All our patients had previous failure to optimized conventional therapy, and the use of vedolizumab in this scenario was warranted in an observational study such as ours. The short follow-up period of the study and the use of “as observed” analysis could have overestimated our numbers, as only patients who achieved specific time points were included as denominators. This could be compensated with the use of NRI analysis. Dose optimization was decided at the physicians’ discretion. Furthermore, the absence of endoscopic scores could influence mucosal healing rates. Despite these limitations, the strength of our study lies in the importance to represent one of the first datasets with vedolizumab used exclusively in mild-to-moderate CD patients who were naïve to biological agents. Our data may help to position vedolizumab in CD treatment algorithms globally.

## Conclusions

In summary, vedolizumab was effective in the management of patients with mild-to-moderate CD as the first biological agent, with a remission rate of 78.8% after 1 year. Mucosal healing was observed in 62.3% of patients and major abdominal surgery was needed in only 4.5% of patients. Rates of primary nonresponse, secondary loss of response, and drug optimization were like other international real-world studies. Discontinuation of vedolizumab occurred in 21.2% of cases over 53.2 months. The safety profile was similar to pivotal trials and international real-world studies. This is one of the first international studies focused on the use of vedolizumab as a first-line biological treatment option in clinical practice in mild-to-moderate CD. Further studies with larger sample sizes and prospective designs are warranted to confirm our findings in this specific population.

## Data Availability

The data underlying this article will be shared on reasonable request to the corresponding author.
